# P2X7 Receptor in the Management of Energy Homeostasis: Implications for Obesity, Dyslipidemia, and Insulin Resistance

**DOI:** 10.3389/fendo.2020.00199

**Published:** 2020-05-12

**Authors:** Roberto Coccurello, Cinzia Volonté

**Affiliations:** ^1^Institute for Complex System (ISC), National Research Council (CNR), Rome, Italy; ^2^Preclinical Neuroscience, European Center for Brain Research (CERC)/IRCCS Santa Lucia Foundation, Rome, Italy; ^3^Institute for Systems Analysis and Computer Science, National Research Council (CNR), Rome, Italy

**Keywords:** P2X7 receptor, energy metabolism, lipid oxidation, adipose tissue, thermogenesis, skeletal muscle

## Abstract

Whole-body energy metabolism entails the highly regulated balance between food intake, nutrient breakdown, energy generation (ATP), and energy storage for the preservation of vital functions and body mass. Purinergic signaling has attracted increasing attention in the regulatory mechanisms not only for the reverse processes of white adipose tissue lipogenesis and lipolysis, but also for brown adipocyte-dependent thermogenesis and leptin production. This regulatory role has remarkable implications in the handling of body's energy expenditure and energy reservoir. Hence, selected purinergic receptors can play a relevant function in lipid metabolism, endocrine activity, glucose uptake, ATP-dependent increased expression of uncoupling protein 1, and browning of adipose tissue. Indeed, purinergic P2 receptors regulate adipogenesis and lipid metabolism and are involved in adipogenic differentiation. In particular, the ionotropic ATP-activated P2X7 subtype is involved in fat distribution, as well as in the modulation of inflammatory pathways in white adipose tissue. Within this context, very recent evidence has established a direct function of P2X7 in energy metabolism. Specifically, either genetic deletion (P2X7 knockout mice) or subchronic pharmacological inhibition of the receptor produces a decrease of whole-body energy expenditure and, concurrently, an increase of carbohydrate oxidation. As further evidence, lipid accumulation, increased fat mass distribution, and weight gain are reported in P2X7-depleted mice. Conversely, the stimulation of P2X7 enhances energy expenditure. Altogether, this knowledge supports the role of P2X7 signaling in the fight against obesity and insulin resistance, as well as in the promotion of adaptive thermogenesis.

## Introduction

At difference with the primary function of intracellular nucleotides that provides the energy supply for cell viability and survival, extracellular nucleotides such as ATP can act as signaling molecules when released into the extracellular compartment. By means of their capacity to use extracellular ATP, adenosine, and other nucleotides and nucleosides for triggering intracellular signal transduction mechanisms, the purinergic receptors have a distinctive role also in the regulation of intracellular metabolism and energy homeostasis (1–3). Basically, there are two wide families of purinergic receptors, the adenosine-binding (P1) and the ATP-binding (P2) subtypes that are, in turn, categorized in P2X ion channels and P2Y G protein–coupled receptors (4). Respectively seven and eight different subtypes have been identified for P2Xs (P2X1–7) and P2Ys (P2Y1, 2, 4, 6, 11–14) receptors, all possessing different binding affinities depending on the various nucleotides (4).

## P2x Receptors, Latent Inflammation, And Obesity-Associated Insulin Resistance

After the discovery by Geoffrey Burnstock of ATP as a noncanonical extracellular mediator (5), accumulating evidence corroborated the idea that ATP being constitutively released by many different cell types allows for cell-to-cell communication. Due to the pleiotropic functional role of ATP, P2X, and P2Y receptors are involved also in a great number of disease conditions. For instance, intense investigation is focused on the therapeutic potential of P2X in cardiovascular diseases (e.g., heart failure, ischemia) (6), cancer (7), neuropathic and inflammatory pain (8), neurodegenerative diseases (9), and muscle and bone disorders (10, 11). Within the context of these diseases, it is also relevant that purinergic receptors are distributed not only in neurons, but also in astrocytes, oligodendrocytes, and immunocompetent microglia (12–14).

In addition to trophic support (15), ATP can also act as a danger signal upon tissue injury or cell damage, by triggering the consecutive phases of inflammatory insurgence and resolution, as well as immune system activation by regulation of T lymphocyte proliferation, T-helper 1 production, and macrophage chemotaxis (16–19). A major common feature underlying systemic inflammation in which P2X receptors are known to play a pathogenic role is altered regulation of energy homeostasis (20, 21). Moreover, there is now large consensus about the role of chronic “low-grade systemic inflammation” as a common factor connecting aging, neurodegenerative diseases, diabetes, and metabolic syndrome (22–24). By definition, obesity is a low-grade systemic inflammatory disease where excessive/uncontrolled energy intake coexists with insufficient energy expenditure (EE) causing abnormal fat accumulation with expansion of adipose tissue (AT) and energy stores (25). It is now accepted that the study of excessive white AT storage (i.e., adiposity) is of major importance for the comprehension of maladaptive chronic inflammatory response and the generation of low-grade systemic inflammation and obesity comorbidities, such as type 2 diabetes, dyslipidemia, cardiovascular disease, and neurodegenerative diseases (26–28). Indeed, over the last decade, the concept of AT as inert energy reservoir has been finally replaced by the notion of endocrine organ, with the identification of a growing number of AT-secreted hormonal factors or adipokines involved in the control of energy homeostasis (29). In parallel, mounting evidence has helped to clarify that adipocytes hypertrophy and aberrant secretory activity involving inflammatory adipokines, such as tumor necrosis factor α (TNF-α) and interleukin 6 (IL-6), are significantly associated with recruitment, infiltration, and accumulation of B and T lymphocytes and macrophages into the white AT and development of insulin resistance (30, 31). On the other hand, before the occurrence of insulin resistance, several mechanisms, such as early activation of proinflammatory and anti-inflammatory immune cells, release of adipocyte-derived cytokines, and secretion of lipolytic hormones (e.g., leptin), can activate the sympathetic nervous system and trigger brown AT-mediated thermogenic responses (32, 33), thus limiting white AT accumulation. However, in the case of protracted positive energy balance, adaptive inflammation and acute immune activation become inadequate responses, and white AT enlargement will produce the alteration of fat distribution, ectopic lipid (i.e., triglycerides) deposition, dyslipidemia, and insulin resistance (34).

Both P2X and P2Y receptors have been found to be regulators of adipogenesis and adipocyte differentiation from bone marrow—and AT-derived mesenchymal stromal cells. In particular, P2Y1, P2Y4, P2Y14, and P2X6 receptors are involved in adipogenic differentiation (35, 36), whereas P2Y2 and P2Y13 receptors have been described in bone marrow–derived adipocyte differentiation (37, 38) (Table 1). The present survey on the role of purinergic signaling in obesity and insulin resistance will be focused on the pleiotropic P2X7 receptor subtype (41) for its major involvement not only in the modulation of fat distribution and inflammation in white AT (42), but also in energy metabolism and nonshivering thermogenesis (39, 40).

**Table 1:** Role of P2 receptors in adipogenic functions and energy metabolism.

**Function**	**Receptor**	**References**
Adipogenic differentiation	P2Y1	(36)
	P2Y4	(35, 36)
	P2Y14	(35)
	P2X6	(35)
Bone marrow–derived adipocyte differentiation	P2Y2	(37) (38)
	P2Y13	
Energy metabolism, thermogenesis, substrate oxidation	P2X7	(39) (40)

## P2x7: From Inflammation Gatekeeping To Energy Metabolism

As other components of the P2X receptor family, also the P2X7 member is largely expressed on immune cells such as B and T lymphocytes, macrophages/microglia, mast cells, and natural killer cells (43), thus contributing to the orchestration of innate and adaptive immune responses (44). Because ATP is also constitutively and/or passively released upon tissue injury or cell damage, the ATP-mediated signaling downstream from activation of P2X7 receptor (possessing a higher Kb for ATP with respect to the other P2 receptors) is often related to the damage- or pathogen-associated molecular pattern molecules and inflammasome formation [e.g., nucleotide-binding oligomerization domain, leucine rich repeat and pyrin domain containing protein 3 (NLRP3)] (43, 45). For this reason, the P2X7 is of major importance for host defense, being considered a sensor of danger signals (46, 47). Indeed, the activation of P2X7 is associated with a remarkable number of inflammatory clinical conditions, including systemic lupus erythematosus and rheumatoid arthritis (48), cancer (49), cardiovascular diseases (50), liver disease (51), colitis and inflammatory bowel disease (52, 53), pain development (54), epilepsy (55), and depression (56).

The main liability of P2X7 in AT inflammation and obesity was at first suggested by the fact that visceral and subcutaneous AT expresses functional P2X7 receptors (both mRNA and protein) and by the notion that such expression is higher in subjects with metabolic syndrome (57). Remarkably, stimulation of isolated adipocytes with the prototypic P2X7 agonist 3′-O-(4-benzoyl)benzoyl ATP (BzATP) triggers the production of IL-6, TNF-α, and plasminogen activator inhibitor 1 (57). Moreover, the exacerbation of the inflammatory status and formation of the P2X7-NLRP3 inflammasome complex have been reported in perivascular AT of subjects with heavy smoking habits (58), in which the overactivation of P2X7 was also associated with higher IL-1β and IL-18 plasma levels. Indeed, P2X7 activation is a potent trigger of IL-1β release and a major cause of innate immune cells activation, proinflammatory activity, and chronic inflammatory diseases (59). However, the same activation of P2X7 is also involved in the release of the anti-inflammatory IL-1 receptor antagonist (IL-1Ra), for instance, in macrophages (60). Of note, it has been shown that an increase of IL-1Ra may provide a protective potential against pancreatic beta cell damage and improve glucose homeostasis in patients with type 2 diabetes, whereas IL-1Ra pancreatic deletion can impair glucose tolerance in mice (61). In agreement with the role of IL-1Ra in glucose homeostasis and beta cell function, the targeted deletion of pancreatic IL-1Ra in mice has been reported to decrease glucose-stimulated insulin secretion and induce glucose intolerance (62). In particular, the study of P2X7-mediated control over IL-1Ra secretion in both lean and obese diabetic patients has helped to disclose a potential regulatory function exerted by PX7 activation on pancreatic beta cell function (63). Moreover, pancreatic IL-1Ra resulted downregulated in beta cell islets of diabetic patients, and P2X7 knockout mice show reduced IL-1Ra secretory capacity, hyperglycemia, glucose intolerance, and impaired beta cell compensation in response to high-sucrose diet (63). Recently, higher IL-1Ra serum levels and improved beta cell function have been found in diabetic patients bearing a polymorphism of the P2X7 gene [i.e., 1,068 G>A, single nucleotide polymorphism (SNP)], although in the lack of significant improvement of glycemic control (64). Together, while the functional association between P2X7-mediated regulation of IL-1Ra secretion and disruption of beta cell function and glucose homeostasis awaits further experimental confirmation, the interplay between IL-1β and the P2X7R is “solid as rock”(65).

Persistent exposure to high-fat diet (HFD) is a well-known experimental model of metabolic syndrome, obesity, and type 2 diabetes, which are relevant risk factors for the increasing incidence of chronic kidney disease (66). High-fat diet–induced renal inflammation involves the P2X7 receptor via the activation of NLRP3 inflammasome, whereas reduced kidney damage, inflammation, and decreased NLRP3 upregulation are observed in mice genetically depleted of the receptor (67). Moreover, while renal P2X7 expression is enhanced in diabetic patients and is associated with damage of glomerular filtration and increased fibrosis, the pharmacological blockade of P2X7 receptor reduces renal macrophage accumulation in experimental diabetic nephropathy (68). It should be noted that P2X7 receptors are expressed by different types of pancreatic cells such as alpha and beta cells, and multiple evidence corroborates the regulatory action exerted by the P2X7 on pancreatic stellate cell proliferation, insulin secretion, and involvement in type 2 diabetes pathogenesis (69–71). Also, the study of P2X7 polymorphisms further demonstrates that P2X7 hypofunction dysregulates glucose homeostasis with reduced insulin sensitivity and compromised glucose tolerance (72). Accordingly, as recently shown (71), P2X7 receptors are activated by glucose elevation, and through their expression on beta cells, they are able to regulate Ca^2+^ signaling, cell proliferation, and insulin secretion. As reported recently, the P2X7 receptor of the beta cells line INS-1E plays a functional role in the regulation of glucose-dependent ATP release. The results prompt for a model according to which glucose is metabolized to ATP after its entry in beta cells, and ATP is also released via pannexin 1 channels under the cooperative regulation of P2X7-dependent modulation of Ca^2+^ influx and potentiation of insulin secretion (71). Within the same line of investigation, it has been observed that P2X7 stimulation induces internalization of GLUT2 in intestinal epithelial cell line (73), and mice lacking P2X7 show upregulation of GLUT2 expression at enterocyte level (74). As a consequence, the authors have found that blood glucose is increased in P2X7 knockout together with hypercholesterolemia, hypertriglyceridemia, and insulin resistance (74).

As for HFD-induced renal inflammation and diabetes-associated renal failure, diabetic retinopathy is exacerbated by sustained P2X7 signaling causing proinflammatory TNF-α and IL-1β release and apoptosis of retinal microvessels (75), which can be limited by P2X7 pharmacological blockade (76). In the context of type 2 diabetes and pathogenesis of insulin resistance, P2X7 activation behaves like a double-edged sword, which may have beneficial effects on glucose homeostasis and pancreatic islet function (71), while in conditions of overnutrition (77, 78) and exposure to obesogenic environments (79), a sustained P2X7 activation concurs to weight gain, hyperglycemia, AT inflammation, and excess of circulating free fatty acids (FFAs).

Indeed, changes of dietary patterns involving the overconsumption of high-energy-dense diets and low-quality foods represent the current nutritional scenario, at least for Western people and developing countries. In such a scenario, it becomes of great relevance to understand the role of P2X7 not only on the impact of nutritional overload, AT enlargement, ectopic fat deposition, and adipose inflammation for the development of insulin resistance, but also its role on defective thermogenesis in obesity.

## Control of Energy Expenditure And P2x7 Signaling

Among the several different hypotheses that have been proposed to explain body mass regulation (e.g., the dual intervention point model) (80), there is the idea that body weight is regularly adjusted and preserved according to a given set point, which results from the balance between energy intake and EE. Because the active defense of body weight set point is constantly threatened by both living in an obesogenic environment and tendency of human genome to store energy as fat, the restriction of energy intake and the increase of EE are the only options to fight body weight gain, adiposity accrual, and obesity. As three major components, EE includes the basal metabolic rate that keeps internal temperature and vital functions, physical activity, and the dissipation of cell energy via heat production (i.e., thermogenesis). It is known that, in addition to white adipocytes where extra calories are stored as triglycerides and released as FFAs, there are also brown and beige/brite adipocytes that are required for adaptive thermogenesis (81–83). The brown AT is highly vascularized and rich in mitochondria and is able to burn energy and generate heat by the activation of the uncoupling protein 1 (UCP1), which “uncouple” mitochondrial respiration and electron transport chain from ATP generation. Because the energy produced by the uncoupling of respiration from ATP production is dissipated as heat into a futile cycle (i.e., proton leak), the brown AT is the major responsible of adaptive or nonshivering thermogenesis. Although UCP1 is mostly brown AT-restricted, low levels of UCP1 are also present in beige/brite adipocytes that are localized within the white AT and exhibit, upon stimulation, inducible thermogenic capacity. Notably, cold exposure, sympathetic nervous system activation, noradrenaline release, binding to β3-adrenergic receptors, prostaglandins, fibroblast growth factor 21, as well as drugs such as thiazolidinediones, may trigger the differentiation from white to brown-like beige adipocytes (83–85). Of importance for thermogenesis in humans, in large adult mammals and not only in rodents, a scattered accumulation of brown adipocytes can still be identified in the neck, in the supraclavicular area, and in paravertebral and perirenal AT (86–88).

**Figure 1:**
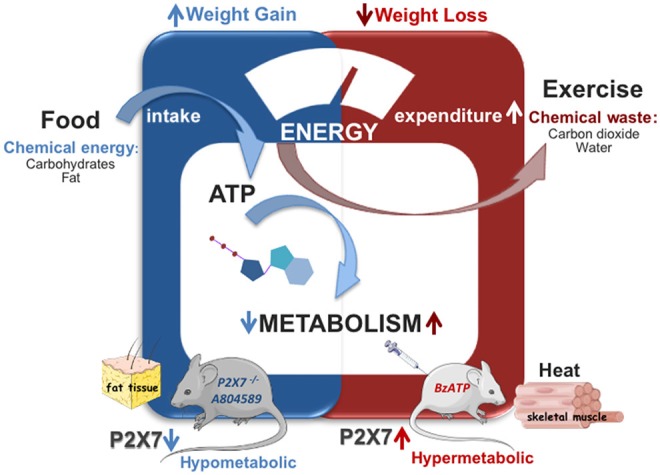
Role of P2X7 in energy metabolism. Genetic deletion or subchronic pharamacological inhibition of P2X7 (A804589) decreases the whole body energy expenditure causing weight gain without affecting food intake (in blue). Stimulation of P2X7 by BzATP enhances energy expenditure and fatty acid oxidation causing weight loss and heat production in mice (in red).

Adaptive thermogenesis is described by the capacity of UCP1 inside the inner mitochondrial membrane to dissipate the electrochemical proton (H^+^) gradient generated by the electron transport chain and to release energy as heat, a mechanism identified 25 years ago (89) and now widely accepted. Uncoupling protein 1 can be activated by the long-chain fatty acids released in brown adipocytes via adipose triglyceride lipase-dependent hydrolysis of triglycerides (90, 91). Recently, UCP1 currents in the inner mitochondrial membrane have been studied by a patch-clamp technique demonstrating that long-chain fatty acids are not only required for UCP1-mediated H^+^ transport activity, but they are also “anchored” in a substrate-like fashion to UCP1 substrates, thus allowing to carry H^+^ across UCP1 (92). Of note, while UCP1 is activated by FFAs, there is also evidence that UCP1 is inhibited by purine nucleotides and, mostly, by ATP-mediated binding on the cytosolic portion of UCP1 that hampers the kinetics of UCP1 translocation (93, 94). Considering the role of P2X7 as sensor of the extracellular ATP environment (being activated by higher ATP concentrations with respect to other P2X receptors) and that loss of P2X7 function in mice disrupts adipocyte distribution inducing adipocyte hyperplasia and lipid ectopic depots (42), a possible opposite impact of loss- or gain-of-function of P2X7 in whole-body energy metabolism has been hypothesized. Indeed, a recent work has demonstrated that genetic depletion and, to a lesser extent, also pharmacological inhibition of P2X7 elicit a pronounced decrease of the whole-body EE and metabolic rate in mice, with a significant increase of the respiratory exchange ratio, thus indicating a prevalent increase of carbohydrate oxidation (39). The relative sparing of fatty acid storage and concomitant defective energy homeostasis were also associated to body weight gain (39), thus generating lipid accumulation, increased fat mass distribution, and, ultimately, weight gain that is also reported in P2X7-depleted mice (42). In line with these results, under standard nutritional conditions, both metabolic rate as measured in terms of O_2_ intake and heat production were significantly increased by subchronic administration of BzATP for seven consecutive days (40). Notably, the increase of metabolic rate/EE was generated also in the lack of motor activity, as detected during the overall resting period recorded across the entire light–dark cycle. Moreover, because the enhancement of EE was not attributable to changes of food intake, it was possible to rule out the potential contribution of diet-induced thermogenesis. On the other hand, because respiratory exchange ratio was decreased by subchronic BzATP administration, the activation of P2X7 produced a significant shift in the level of nutrient substrate utilization, and in particular of lipid consumption over other food energy sources (i.e., carbohydrates and proteins). Being simultaneously consumed more O_2_ without evident alteration of motor behavior, an increase of fatty acid oxidation and a prevalent use of lipids as energy source after P2X7 receptor stimulation, was demonstrated. Providing further confirmation, the preadministration of the specific P2X7 antagonist A804589 delayed and attenuated the increase in O_2_ consumption and EE induced by BzATP-induced potentiation of P2X7 activity (40) (Figure 1, Table 1). Thus, these data support the notion that an opposite regulation of P2X7 function (namely, suppression vs potentiation) is able to produce antithetical effects on whole-body energy metabolism, being the EE reduced by P2X7 suppression and boosted by P2X7 potentiation. Accordingly, while lipid oxidation is decreased by suppression of P2X7 function, fatty acids are more selectively oxidized by the stimulation of P2X7 function (39, 40).

## Muscle Machinery, Fatty Acids Oxidation, And P2x7 Signaling

As one of the largest tissue accounting for ~50% of body mass, the skeletal muscle is a major determinant of the whole-body metabolic rate (95), possessing a remarkable capacity to rapidly shift from carbohydrates to fatty acids utilization in response to increasing energy demand and intensity of physical exercise (96–98). As a consequence, generation of reactive oxygen species (ROS) is augmented in skeletal muscles as part of a physiological response to exercise, adaptation to increased workload, and optimization of the contractile capacity (99). In particular, in the muscle gastrocnemius the physical exercise specifically stimulates the mRNA levels of NADPH oxidase 2 (NOX2) (100), a superoxide-generating enzyme and major source of ROS under resting and contractile muscle conditions (101). Importantly, BzATP-induced activation of P2X7 increases the content of the glycoprotein gp91phox (a component of the membrane bound NADPH oxidase complex) in the gastrocnemius (possessing both fast- and slow-twitch fibers) and tibialis anterior (with 95% fast-twitch fibers) muscles, thus indicating that P2X7 activation might have a role in the contraction-induced intracellular signaling mediated by nonmitochondrial ROS sources such as NOX2 (40). We do not exclude that the BzATP-induced activation of P2X7 might mimic the increase of oxygen consumption (102, 103) and the NOX2-dependent redox adaptation that occur in mixed and fast-twitch glycolytic fiber types (104) after high-intensity training and endurance exercise. Under this perspective, the sustained activation of P2X7 might cause a remodeling of fatigue-susceptible muscles (i.e., type II fibers) and reprogramming of the global muscle gene expression toward a slower, more oxidative transcription program.

Skeletal muscle exhibits shivering and adaptive thermogenic capacity, as well as the ability to increase EE as a function of different types of physical exercise, adjusting the intensity of contractile activity and metabolic pathways to the changing energy requests (i.e., by shifting from carbohydrates to prevalent fatty acids utilization). These remarkable features, combined with the well-known possibility to breakdown almost 80% of the insulin-stimulated glucose uptake, make the skeletal muscle a specialized tissue to manage insulin sensitivity and, whenever defective, a primary player in type 2 diabetes pathogenesis. It is known that P2X7 activation triggers the opening of cations permeable channels allowing a considerable mobilization of intracellular Ca^2+^ (105) and the increase of mitochondrial Ca^2+^ and cellular ATP levels (106). Interestingly, within the context of Ca^2+^ handling and preservation of muscle physiology, the activity of sarco(endo)plasmic reticulum Ca^2+^ATPase (SERCA) provides a key contribution not only to the maintenance of intracellular Ca^2+^ homeostasis, but also to the nonshivering thermogenesis. By subserving the removal of cytosolic Ca^2+^ and its reuptake into the sarcoplasmic reticulum lumen (107), the SERCA pump contributes to Ca^2+^ handling and muscle contraction–relaxation cycle (108). As a result, a protracted inhibition of SERCA activity may lead to cytosolic Ca^2+^ overload, as observed in defective muscle regeneration and muscular dystrophies (109, 110). Indeed, excessive increase of intracellular Ca^2+^ is an early pathogenetic event in the progression of Duchenne muscular dystrophy (109–112), and P2X7 receptor seems to have a role in muscular dystrophies (113, 114). Thus, the excessive levels of extracellular ATP observed in dystrophic muscle might contribute to the overactivation of the P2X7 receptor and then to abnormal intracellular Ca^2+^ homeostasis and chronic inflammation (113–115). Because sarcolipin binding to SERCA is facilitated by high cytosolic Ca^2+^ (116), there is the possibility that P2X7 overactivation and increase of intracellular Ca^2+^ may promote sarcolipin binding to SERCA and thus increase nonshivering muscle thermogenesis.

The most part of whole-body adaptive thermogenesis that occurs in skeletal muscle cannot rely on UCP1 (not expressed in muscle), but on the analog UCP3 that is prevalently expressed in both skeletal muscle and brown AT (117). Uncoupling protein 3 is activated by FFAs and can be inhibited by the direct competition between FFAs and purine nucleotides (118). The expression of UCP3 in muscle appears regulated by the levels of fatty acid oxidation occurring during prolonged physical activity (119), fasting (120), or under HFD regimen (121), supporting the concept of UCP3 as functionally involved in muscle fatty acid transport, beta-oxidation (122), and protection against HFD-induced insulin resistance (123). Although muscle UCP3 does not contribute to brown AT-mediated thermogenesis (124, 125), there is nevertheless evidence that in the lack of UCP3 both the thermogenic response to sympathomimetic drugs and lipopolysaccharide challenge are blunted (126, 127), thus supporting the notion that UCP3 may be important in amplifying brown AT thermogenesis and promoting skeletal muscle thermogenic activity and/or fatty acid oxidation (127, 128). Most importantly, it should be underlined the protective mechanism exerted by UCP3 against mitochondrial fatty acid accumulation and fatty acid–induced damage of mitochondrial oxidative capacity (129–131). Further support to this aspect comes from experiments on UCP3 knockout mice in which an increase in mitochondrial ROS production is observed together with reduced uncoupling activity but in the absence of body weight alterations or cold-induced thermogenesis (132, 133). Under this regard, the result that direct P2X7 activation increases fatty acid oxidation and reduces body weight might also suggest a potential UCP3-mediated facilitatory action of the receptor.

## Conclusive Discussion

In the present survey, we have highlighted the major involvement of P2X7 in determining and regulating fat distribution, white AT inflammation, energy metabolism, and nonshivering thermogenesis. Indeed, functional P2X7 receptors are found expressed in visceral and subcutaneous AT, and the alteration of P2X7 function greatly contributes to AT inflammation and adiposity.

Next, we reported recent evidence that modulation of P2X7 can change whole-body EE, also determining the rate of macronutrient oxidation and the adjustment of fuel selection (i.e., carbohydrate vs lipid) to achieve body weight regulation. In particular, we have reported that BzATP-induced activation of P2X7 increases EE and induces NOX2 expression in gastrocnemius and in fast-twitch tibialis anterior. Because balanced levels of NOX2 and ROS are viewed as mediators of glucose transport in muscle (134), we suggest that activation of P2X7 function may not only increase fatty acid oxidation and EE and decrease body weight, but also possibly reduce the susceptibility to insulin resistance under high-fat feeding and/or during the dysregulation of the muscle P2X7/Ca^2+^/ UCP3/sarcolipin-SERCA axis.

Because selective overexpression of UCP3 in muscle has been found to act as exercise mimetic not only by increasing fatty acid oxidation and EE (135), but also by modulating the mutual interplay between ROS generation and fatty acid oxidation (133), it might become important to next improve our knowledge about the ability of P2X7 to shape the metabolic signature of muscle fibers mimicking high-intensity training and endurance exercise (40, 102). However, given the “double-edged sword” nature of P2X7 (136), caution must still be recommended because continuous or excessive stimulation of P2X7 could exacerbate endoplasmic reticulum stress that has been recently recognized to be mechanistically involved in the decrease of brown AT-dependent EE and obesity progression (137).

## Author Contributions

RC conceived the study. RC and CV wrote the manuscript.

## Conflict of Interest

The authors declare that the research was conducted in the absence of any commercial or financial relationships that could be construed as a potential conflict of interest.

## Abbreviations

AT: Adipose tissue
BzATP: 3′-O-(4-Benzoyl)benzoyl ATP
EE: energy expenditure
FFAs: free fatty acids
HFD: high-fat diet
IL: interleukin
IL-1Ra: interleukin-1 receptor antagonist
NLRP3: nucleotide-binding oligomerization domain, leucine rich repeat and pyrin domain containing protein 3
ROS: reactive oxygen species
SERCA: sarco(endo)plasmic reticulum Ca^2+^ATPase
TNF-α: tumor necrosis factor α
UCP1: uncoupling protein 1
UCP3: uncoupling protein 3.
